# Julia Greer answers questions about additive manufacturing

**DOI:** 10.1038/s41467-020-17723-2

**Published:** 2020-08-17

## Abstract

Professor Julia R Greer is a materials scientist at the California Institute of Technology. Her group focuses on designing, fabricating and characterising micro- and nano-architected materials using 3D lithography, nanofabrication, and additive manufacturing (AM) techniques for a multitude of applications ranging from biological devices to damage-tolerant fabrics.

Tell us a little bit about you and what sparked your interest in additive manufacturing.

When I was a graduate student, my PhD research was about the mechanical properties of nanomaterials. It was a very fundamental study where I made very small nanostructures, deformed them, and measured their strength. But in the back of my mind, I was always wondering: these structures are a little bit like Lego, so can we do something useful with them, maybe give them fun or interesting shapes? It’s the same concept as the trusses making up Eiffel tower, or using tiny building blocks to build a crane.

Before tenure, you are not really encouraged to think outside the box. So it is really only after I got tenure that I felt like I was in a position to pursue this idea. It was a leap of faith, which coincided with the development of a technique called two-photon lithography (TPL). I went to Germany to visit a company called Nanoscribe, which spun out of the Karlsruhe Institute of Technology. They demonstrated the capabilities of the technique and I was sold: they even printed a little Statue of Liberty and a “Welcome Julia” sign for my visit! I realised that this was the instrument that was going to enable me to achieve the scientific dream that I had, which was to use these nanomaterials to make something larger out of these small, tiny things, and together with a colleague, we bought the countryʼs (USA) second Nanoscribe instrument.

One of the ways in which I am really fortunate is that Caltech students are brilliant, they educate and inspire me: one of the great things that came out of getting acquainted with the Nanoscribe together is all the things that are not spelled out in the manual. We explored what happens when you coat the structure you print with another material, or when you modify the resin, and when you have a cascade of chemical reactions… . This is what catalysed my transition to additive manufacturing. Additive manufacturing was a springboard for us to explore what else could be done with these materials. There is a lot of potential out there! We were pioneers in terms of 3D architected nanomaterials and in the development of different synthesis techniques (for example to make smart textiles, or materials that are simultaneously ultralightweight and ultrastiff). Today, my group’s niche is in the discovery and demonstration of these unexpected phenomena in 3D nano-architectures made of a broad range of materials.

How has additive manufacturing changed your field? Is there anything specific that it has enabled or made possible? Tell us how it impacted your research.

Additive manufacturing has hugely impacted my field. It is exploding right now, and is a topic of conversation and discussion at national agencies and the National Academies: for example,  additive and digital manufacturing have been highlighted by the US Department of Energy and the National Materials and Manufacturing Board. There is no conference these days without an additive manufacturing symposium! It is enabling many research groups and allowing for explorations that wouldn’t be possible without it: some examples are multi-functional, reconfigurable smart materials and devices, new device physics, materials research….

There has always been a duality between material and structure, and additive manufacturing enables us to couple these properties. We can envision a day where we have a backpack with supplies and we can use natural resources and the natural habitat to set up a little 3D-printer and create power and energy sources and whatever else we may need: solid state batteries, fuel cells… Additive manufacturing allows us to dream big, which is what I like doing. While I’m not sure of the timeframe, it really paves the way for a future world where everything is going to be done by design according to utility.

What does additive manufacturing have to do to be more widely adopted? Are there any specific hurdles it has to overcome?

There are currently two big gaps: one is a lack of computational capabilities that are able to accurately describe and predict properties, and another is scalability.

We currently have computational models that are high fidelity but are expensive in terms of time and not particularly generalisable or ones that are less computationally expensive but canʼt capture the physics of the additive manufacturing process. Nearly all models are idealised and lack the ability to account for defects or to accurately predict or describe properties of materials that contain some defects. What we need is for models not only to capture the processing parameters and properties that emerge during the additive manufacturing process, but also to be able to make in-process decisions to adjust the final product. We are effectively looking for in situ diagnostic capabilities as well as the ability to process the acquired data in real time. Today, we just don’t have reliable models, and the bottlenecks are computational bandwidth and our predictive capabilities. Using machine learning techniques and genetic algorithms can be one way to accommodate defect detection and accommodate large amounts of data, which will then give rise to the ability to predict defects in a reliable way. Navigating this infinite parameter space is the real challenge: Once we can do that and successfully achieve in-process decision making capabilities, developing and refining the manufacturing techniques will be straightforward.

The other issue is scalability: how can production be stepped up while retaining the desired properties for these materials to be inserted in useful applications? To this end, I’ve co-founded a company specifically focused on scalability that makes smart textiles: from very porous filters to ultralightweight mechanically resilient sheets. We had to deviate from two-photon lithography as it is not amenable to mass production, and develop a different method for patterning large areas.

Looking forward: where do you see additive manufacturing going next?

Looking at the next 5 years, more and more real companies are looking into 3D printing, which is one aspect of additive manufacturing. 3D-printing is becoming mainstream technology for component development where cost isn’t a factor: for example, in biomedical, and space and airborne applications. I believe these niche applications where either the current cost of manufacturing is high or the economic aspects of the produced part are less important will be the first ones to truly adopt additive manufacturing and make it proliferate. You could argue that today we are able to print fully degradable plastic bags using polylactic acid, for example, but the reality is that it is impossible to compete with the economics of current high-density polyethylene (HDPE)/low-density polyethylene (LDPE) plastic bag production.

The manufacturing community is currently focussing on scalability and the development of computational techniques, both being hurdles I talked about above. Current buzzing topics to apply to additive manufacturing are machine learning techniques and genetic algorithms, which have previously been developed in other contexts.

For you, is there a difference between additive manufacturing and 3D printing? If they are not equivalent, how do they differ?

They are different! 3D-printing is layer-by-layer shaping of a three-dimensional structure. Additive manufacturing is more complex than that, involving chemical synthesis and clever algorithms, which ulitmately leverage on material and structural effects. The materials that can be produced using additive manufacturing are far from equilibrium, and it wouldn’t be possible to create and shape these configurations without additive manufacturing. Understanding how a material behaves at each relevant  lengthscale in a structure where everything is essentially chemically derived ins a challenge! Some of these may be detrimental, and others become useful features. There is so much more to learn!

